# Studying Ancient Anthropogenic Impacts on Current Floral Biodiversity in the Southern Levant as reflected by the Philistine Migration

**DOI:** 10.1038/srep13308

**Published:** 2015-08-25

**Authors:** Suembikya Frumin, Aren M. Maeir, Liora Kolska Horwitz, Ehud Weiss

**Affiliations:** 1 The Institute of Archaeology, Department of Land of Israel Studies and Archaeology, Bar-Ilan University, Ramat-Gan 52900, Israel; 2 National Natural History Collections, Faculty of Mathematics and Sciences, The Hebrew University of Jerusalem, Jerusalem 91904, Israel

## Abstract

Human migrations across geographic boundaries can facilitate the introduction of new husbandry practices and dispersal of plants and animals, resulting in changes in biodiversity. As previously demonstrated, the 12th century BCE Philistine migration–to the southern Levantine littoral, involved the transportation of pigs from Europe, engendering long term genetic displacement of local Near Eastern haplotypes. Building on this, and combining biogeographical methods of Floral List comparisons with archaeological data, we have elucidated the Philistine impact on Southern Levantine floral ecosystems. We demonstrate that previously unexploited local plants were incorporated into the Philistine milieu, and new species were introduced–from Europe, the Aegean, Egypt and Mesopotamia –resulting in the earliest locally cultivated sycamore, cumin, coriander, bay tree and opium poppy. This research has highlighted the impact of past cultures on the formation of floral ecosystems and their long-term effects on contemporary local biological diversity.

Human migrations across geographic boundaries promote the long-distance dispersal of plants and animals, resulting in biological invasions. Together with new techniques of land management that often accompany the migrants, this leads to long term changes in natural biomes. Salient examples include the European conquest of the New World^1^, the Roman occupation of Britain^2^ and dispersion of Neolithic domesticates across the Old World^3^,^4^. Here we propose a novel research approach aiming to study the different anthropogenic impacts on an ecosystem resulting from the advent of an extinct historical culture, the Philistines^5^,^6^–one of the so-called “Sea Peoples”–that appeared in the southern Levantine littoral, after ca. 1,200 BCE. Until quite recently, the accepted view was that the Philistines originated from a single region, most likely somewhere in the Aegean^7^,^8^. Recent research^5^,^6^,^9^ has revised this view and shown that in fact, the Philistine culture is comprised of migrants of multiple foreign origins, including the Aegean, who, when arriving in Canaan, intermingled with local Canaanites. The non-Levantine origin of a substantial portion of the Philistine culture is evidenced by their distinctive architecture, ceramic ware, technologies and ritual activities that point to their diverse and multifaceted origins with different components resembling Aegean, Cypriot, Anatolian, Egyptian and even Southeast European cultures^5^,^6^,^9^.

In contrast to the situation during the previous period, the Late Bronze Age, where most of the foreign components seen in the Southern Levant arrive due to trade connections with various parts of the eastern Mediterranean^10^, it is clear that the majority of the non-local facets found in early Philistine culture appear in the region due to the arrival of migrants from foreign regions^5^,^6^. Following the initial appearance of the Philistine culture, contact with their former homelands–and neighboring cultures—continued so that they maintained a distinctive but “entangled” culture^5^,^6^. In addition to cultural innovations, the Philistines transported pigs (*Sus scrofa*) from Europe into the southern Levantine littoral, facilitating genetic displacement of local Near Eastern haplotypes during the Iron Age^11^. Gradually, many features of the Philistine cultural repertoire spread beyond their settlements and were adopted by other local Levantine populations^5^,^6^.

In this study we focus on floristic changes that are associated with Philistine migration and which serve as a proxy data set with which to measure past anthropogenic-engendered changes in plant and land management. We investigated diachronic and temporal trends in biodiversity by comparing Floral Lists (FLs) of species (Fig. 1; Methods) based on published archaeobotanical reports (Supplementary Information Tables S1 and S2), from periods preceding, during and subsequent to the advent of the Philistines.

Data were compiled for 18 Bronze Age archaeological sites from Israel that pre-dated the Philistines (Early to Late Bronze Ages combined, spanning ca. 3,500-1,180 BCE), as well as 20 Iron Age sites. These include early Iron Age (ca. 1,180-950 BCE) and late Iron Age (ca. 950-586 BCE) sites, divided into those that are generally accepted as Philistine settlements (7 sites) versus non-Philistine (i.e., Canaanite, Israelite, Judahite, Phoenician;15 sites; see Figs 1, 2 and 3; Supplementary Information Table S1 and S2, and Methods). Moreover, in the Iron Age sites of Aphek and Batash there are consecutive strata, representing Philistine and non-Philistine cultural affinities. The Bronze and Iron Age sites in the data base vary in character and function, from large as well as small-scale urban sites, to various kinds and sizes of rural sites. The data represent several decades of careful and continuous collection and analysis of plant remains (including on-site collection by two of the authors), with specific care for the identification of wild plants and crop plants to the species level in an attempt to create a data set that could be used for environmental reconstructions^12^. Moreover, the Bronze Age archaeobotanical data derive from sites that lie in diverse geographic and climatic zones (Fig. 1), and so constitute the full spectrum of local plants that were exploited by pre-Philistine local communities. The large number of sites samples, from various periods and of different types (urban, rural, etc.), enables us to present a comprehensive diachronic and synchronic understanding of plant use in different periods, cultures and ecozones in the ancient Southern Levant. For our analysis of the FLs, we used standardized indices of biodiversity to assess and quantify the patterns of temporal change (Coefficient of Community^13^, CC, and Jaccard similarity coefficient^14^, D_S_).

## Results

Comparison of the combined Bronze Age FL (species n = 178) with the combined Iron Age FL (species n = 269) revealed marked changes in biodiversity between periods (Ds = 0.65, CC = 0.54). Altogether, 149 new species appeared in the region in the Iron Age that were not recorded in the previous Bronze Age sites. The new species encompass synanthropic trees, herbs and plants associated with both dry and wet habitats.

In order to differentiate between the effects of climate change^15^ and cultural change on floral biodiversity within the Iron Age, a comparison was undertaken of Philistine versus non-Philistine FL’s, with early and late Iron Age periods combined (Fig. 3). This comparison revealed unequivocally high indices of variation in Iron Age floral biodiversity between the two Iron Age groups (Ds = 0.73, CC = 0.43). The clear distinctions seen between the FL groups are validated by a Rarefaction test (Supplementary Information SI 3) which shows that sample size is not the source of the observed differences in species diversity.

**Figure 2:**
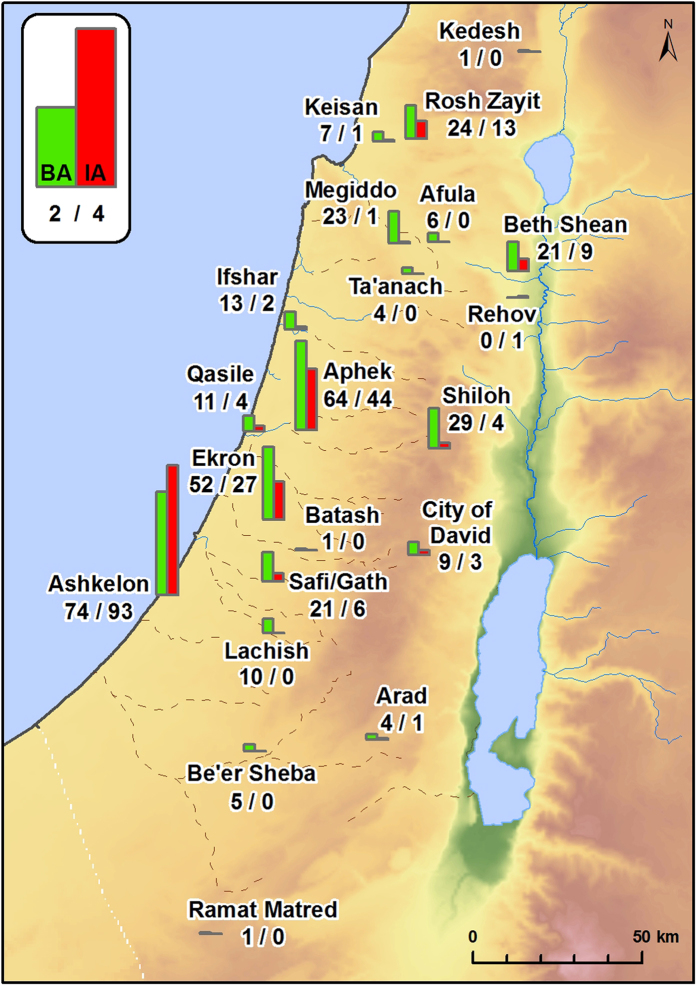
Species turnover between the Bronze and Iron Age at Iron Age sites. Each site is marked by two columns. The green column marks the number of Bronze Age species found in the Iron Age floral list. The red column marks the number of new species in Iron Age sites. Numbers beneath the site name give the absolute numbers of Bronze Age/Iron Age species. Map produced by M. Frumin using ArcGIS for Desktop (ArcMap 10.1), ESRI.

**Figure 3:**
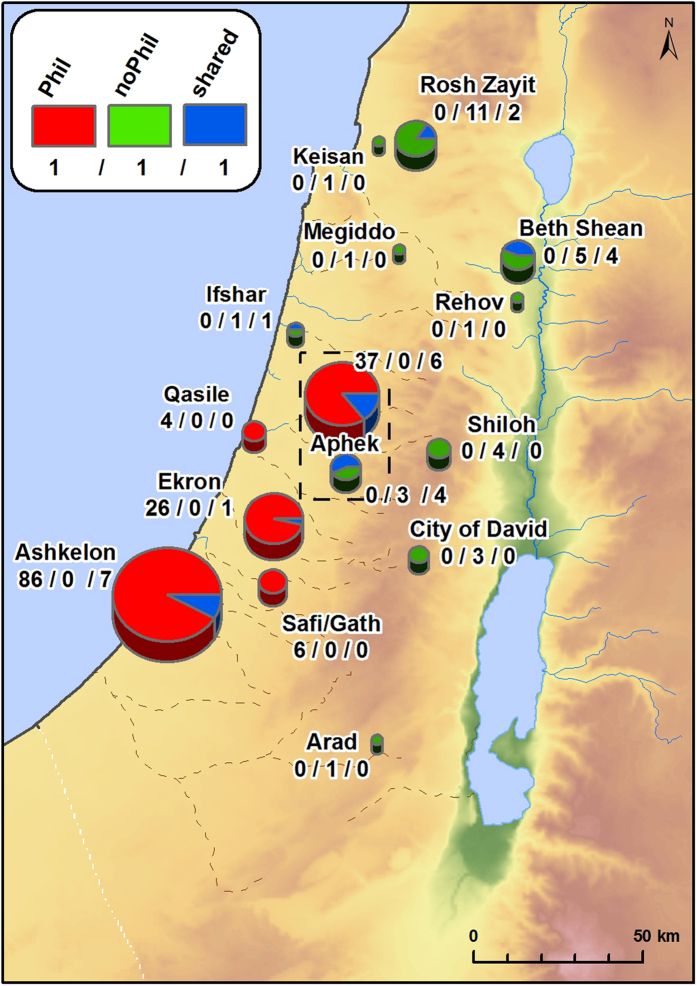
Structure of Iron Age Floral List at each sites. Circle size reflects the total number of new plant species recognized in Iron Age sites. Red indicates new species that appeared only in Philistine Iron Age sites. Green indicates species that appeared only in non-Philistine Iron Age contexts. Blue denotes species shared by Philistine and non-Philistine sites. The three numbers represent the quantity of Philistine species/non-Philistine species/shared species, at a site. Map produced by M. Frumin using ArcGIS for Desktop (ArcMap 10.1), ESRI.

The Philistine FL (species n = 219) includes 113 new taxa recorded only at Philistine sites. That means that, 51.6% of taxa in the Iron Age Philistine FL are new species that were previously unknown in the Bronze Age archaeobotanical record, compared with only 29.5% new species (n = 36) in the non-Philistine FL (species n = 122, Figs 2 and 3). The distinction in FL between non-Philistine and Philistine settlements is confirmed by diachronic trends at the site of Aphek (Fig. 3). At this site, the dominant cultural attributes changed during the Iron Age from non-Philistine (Stratum X11) to Philistine (Strata X10, X9) and back to non-Philistine (Stratum X8)^16^. In the Philistine Iron Age strata, 64% of the species showed continuity with the Bronze Age versus 87% in the non-Philistine strata. In the Philistine Iron Age strata, 29 new species are found versus only 5 in the non-Philistine strata. Moreover, species associated with the Philistine strata do not continue into the non-Philistine strata. It is interesting to note, that there are two new cultivars for Israel that first appear in Iron Age sites in non-Philistine strata, both are known from Iran. These are *Prunus armeniaca* (Apricot, in the City of David^17^) and *Celtis australis* (European nettle tree, at Rehov^18^). Apricot is known so far only from the Iron Age strata from the Iranian site of Bastam^19^, while Nettle tree is present at numerous Iranian sites during the Bronze Age^19^.

The distinct effect of Philistine migration on local floral biodiversity of southern Levant is evidenced in three features that are discussed below.

### (1) Introduced species

**Figure 4:**
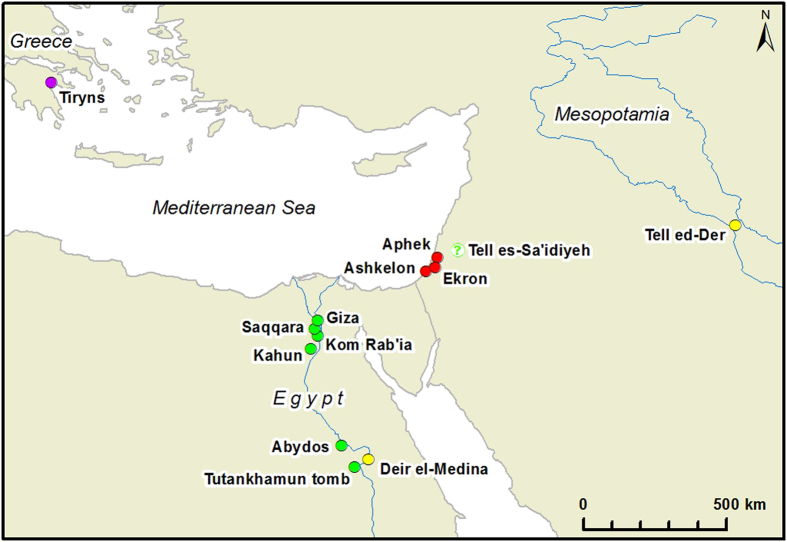
Map of findings of Cumin, Opium poppy and Sycamore during Bronze Age within Eastern Mediterranean and the Near East. Dot color marks species reported from the site: purple–Opium poppy, yellow–Cumin, green–Sycamore. Red dots indicate Iron Age Philistine sites where these plants were found for the first time. Map produced by M. Frumin using ArcGIS for Desktop (ArcMap 10.1), ESRI.

The new synanthropic species introduced by the Philistines (Fig. 4) comprise three cultivars, *Cuminum cyminum* (cumin, found at Aphek, Stratum X10, early Iron Age), *Ficus sycomorus* (sycamore, found at Ashkelon, late Iron Age) and *Papaver somniferum* (opium poppy, found at Ashkelon, late Iron Age). The full biogeographic distribution of cumin and sycamore is still not fully understood, but includes parts of the Eastern Mediterranean, while opium is a cultivar of west European origin^20^. We perceive these taxa as translocated species as today, none of them grow in Israel in the wild but only occur under cultivation^21^.

In the Eastern Mediterranean, the earliest cumin has been identified from Atlit-Yam (northern Israel, Neolithic, ca. 6, 900-6, 300 BCE)^22^, subsequently it was found in New Kingdom Egypt (Deir el-Medina, 18^th^ dynasty, ca. 1,543-1,292 BCE)^23^ and in Mesopotamia (Tell ed-Der, ca. 2,100-1,900 BCE)^19^, and re-appears in Israel only at Iron Age Philistine sites.

Sycamore is an eastern African species, domesticated in Egypt and closely associated with Egypt agriculture since Predynastic times (i.e. Neolithic period, ca. 6,000-3,100 BCE)^20^,^23^. All parts of this tree were found in Egypt in numerous tombs in the Valley of the Kings through the Early, Middle and Late Kingdoms (ca. 3,000-1,000 BCE)^23^ and later probably in Tell es-Sa’idiyeh, Jordan (ca. 2,900 BCE)^24^ (Fig. 4). Outside Egypt, the sycamore findings include so far only timber. The earliest sycamore *timber* was found in Jericho (Neolithic)^25^. The next occurrence is in Beth Shean (Late Bronze Age)^19^, and from the Iron Age onwards, sycamore timber is a common find in Israel^25^. However, the remains of its fruit (rather than wood) first appear, outside Egypt, in Philistine contexts at the site of Ashkelon, and probably represent the exploitation of locally grown trees.

The finding of opium poppy at Philistine Ashkelon is the second earliest evidence of opium seeds (and possible cultivation) of this plant in the Eastern Mediterranean. The earlier example came from Late Bronze Age Greece (Mycenaean Tiryns, ca. 1,200 BCE)^19^, from where some of the Philistines may have originated. In previous periods, particularly the Late Bronze Age, there is evidence of trade in opium into the Eastern Mediterranean, but no signs of local cultivation^26^,^27^. In addition the inscriptions on Sumerian clay tablets dating to the Early Bronze Age that have been previously interpreted as referring to the Opium poppy, are now interpreted as referring to *Punica*, the pomegranate^26^,^28^,^29^. The presence of the seeds and not of opium latex itself in a Philistine site suggests the possibility of local cultivation of the plant in Israel. Thus, our result imply that cumin, sycamore fruit and the opium poppy seeds were first introduced into Israel by the Philistines, from remarkably diverse regions to the north-west and south-west of Israel (Fig. 4).

### (2) Changes in dietary preferences

Among species that are absent in Bronze Age contexts in Israel and which first appeared during the Iron Age in Philistine sites, there are two useful plants species, which occur in the wild in Israel: *Coriandrum sativum* (coriander, from early Iron Age Ashkelon^30^,^31^ and Ekron^31^) and *Laurus nobilis* (bay tree, from late Iron Age Ashkelon)^30^,^32^. Coriander is well-represented in Bronze Age contexts in the Eastern Mediterranean in sites to the north and north-west of Israel, as well as in Egypt (Tutankhamun’s tomb)^19^. Though it is found in a Neolithic site in Israel (Nahal Hemar cave, ca. 6,000 BCE)^33^, it disappears from the region and is not found during the Bronze Age^19^, only to reappear in the Iron Age in Philistine sites. Bay tree *timber* is known in Bronze Age Israel from two sites in the arid south–Arad^25^ and Jericho^25^ –and was apparently transported to these sites, as its natural habitat is the Mediterranean woodland. Bay tree *fruit* is first found in Philistine Ashkelon^32^ suggesting a possible change in plant use, from timber to fruits–and possibly also, of its leaves. These temporal changes can be attributed to changes in dietary preferences among the Philistine peoples who settled in the littoral of the southern Levant. Today, both these plants species grow in Israel in natural habitats and under cultivation^21^.

### (3) Changes in land-use

Ten new synanthropic species first appear in early Iron Age Philistine sites and an additional six in the late Iron Age (Table 1). Many of the new species that appeared with the Philistine culture are members of the Fabaceae (Legume) family, and belong to the genera *Trifolium, Pisum*, *Trigonella, Lathyrus* and *Vicia* that already appeared in the country in Bronze Age FLs. However, 40% of the new species belong to genera that are new and unknown in Bronze Age sites (Table 1). These include *Eragrostis pilosa* (soft lovegrass, early Iron Age), *Portulaca oleracea*, *Raphanus raphanistrum*, *Salsola kali*, *Hyoscyamus albus,* and *Vigna luteola* (species of Purslane, Wild Radish, Saltwort, Henbane and Vigna, appear in the late Iron Age). These new species all relate to synanthropic habitats as invasive weeds and/or as serviceable plants (Henbane, Saltwot, and Wild Radish)^34^, or as culinary species (Vigna, Soft Lovegrass, and Purslane)^20^,^35^, suggesting that their sudden appearance is related to human selection. These findings, namely variation at a genus level with a high level of synanthropy among the new species, indicate changes in land-use, either in agrarian techniques or in habitats exploited by the Philistines. Their impact is still visible on the local Israeli biome, with almost half of the synanthropic species that first appeared with the Philistines in the early Iron Age (n = 41 spp.) still associated with synanthropic habitats throughout Israel today^21^.

## Discussion

To the best of our knowledge, the present study is the first attempt to examine how changes in ancient plant diversity and use can unravel how human migration can impact the formation processes of ancient, as well as current biodiversity. Our results show that despite the shared agrarian base of all Levantine sites during the Bronze and Iron Ages–centered on cultivation of wheat, barley, lentils, grape, fig and olives–the range of exploited plant species changed significantly in the wake of the appearance of the Philistine culture (which includes substantial migrant components). It expanded to include several species which had not been cultivated in the preceding Bronze Age in the Levant along with many synanthropic weeds and wild species. Our results demonstrate that at least three previously undocumented cultivars first appeared in the southern Levant concurrently and in the same geographic locality as the Iron Age Philistine culture. These plants are known in the Bronze Age archaeobotanical data in different regions within the Mediterranean region, but not from Israel. Moreover, the Philistine Floral List differs significantly from those of contemporaneous sites associated with local non-Philistine communities–Canaanites, Israelites, Judahites and Phoenicians. Though climatic and edaphic difference may explain some portion of the floristic distinction observed between the coastal Philistine culture and their inland neighbors, nevertheless, cultural differences appear to be responsible given the discrepancy between FLs from different layers within the site of Aphek. Besides, appearance of apricot and nettle tree in non-Philistine Iron Age strata further support human-engendered changes in plant diversity during the Iron Age. Further investigation of Philistine culture and comparison with different cultures at the same site, or with closely situated settlements within the same geographic region, promise to shed more light on this phenomenon.

Although, our research is based on data retrieved by different archaeological teams, it should be stressed that the observed differences and changes in the floral lists mirror those seen in many facets of the comparison between the Philistine and non-Philistine material cultures^6^,^36^,^37^. Thus, the appearance of the Philistine culture, with its foreign human elements which arrived by migration, was accompanied by translocation of plant and animal species that derive from diverse localities, and together with the introduction of new agrarian technologies led to changes in diet and land-use^38^. This fits in very well with our understanding of the multiple origins of Philistine culture, related mainly to Mediterranean littoral cultures. The introduced Philistine dietary package joins the few, select examples known from antiquity of such extensive translocations of both exotic flora and fauna. The impact of this migration on local biota spanned some 600 years through the Iron Age, indicating multiple introduction events from diverse regions, matching other archaeological evidence for the diverse origins and connections of this culture^6^. The long-term impact of these 12^th^ century BCE floral introductions is still evident today in the Israeli landscape (e.g. *Ficus sycomorus*), and demonstrates the distant and often complex histories and interactions of much of the local synanthropic flora, and the *longue durée* effects manifested in contemporary biodiversity.

## Methods

To test temporal and cultural changes in biodiversity one need a comprehensive data base that allows exploring the patterns and mechanisms of variation. The data should represent the whole area of investigation, through the adjacent time periods. However, the very nature of archaeobotanical data, as all archaeological data is haphazard. Closely situated sites may represent different plant species due to difference in human cultures back in time, but also due to possible diversity in accumulation and conservation processes. Accumulation during winter- or summer crops maturation; destruction of settlement after siege or drought, when all the possible food is finished, or accidental fire that carbonize rich house or settlement enhance variation within the available material. Weeds composition may change with age of cultivated field and succession stage of natural vegetation. Also, the type of excavated material–vessels from granary or waste pit may enhance the difference in species recognized. Thus, spatiotemporal comparison should be applied on as wide as possible floral list, incorporating data from several contemporaneous sites. Hence, we assembled a database of floral taxa (species hereafter, Floral List hereafter FLs) carefully identified by seeds and fruits in all available Israeli excavations published (from 1955 up to mid-2013, Supplementary Information Table S1), spanning the Early, Middle and Late Bronze Ages (which were pooled; 33–13 centuries BCE), the early Iron Age (ca. 1,180-950 BCE) and the late Iron Age (ca. 950-586 BCE). We excluded from analysis taxa described from strata attributed to the transition/boundary period of Late Bronze-Iron Age (e.g. Bet Shean 17A, 20th Dynasty Egyptian) because the main goal of our analyses is to detect the difference between the periods. Thus, our data on plant species from the Bronze Age were collated for 18 different archaeological sites (Supplementary Information Table S1). These represent a wide range of settlements: such as the larger urban sites of Aphek, Arad, Ashdod, Beth Shean, Ekron, Lachish, Megiddo, Safi/Gath, and Ta’anach; small-scale urban sites such as Batash, Jericho, Ifshar, Shiloh, and Qasile; and various types of rural sites such as Afula, Manahat, Nami and Shiqmim^36^. The Iron Age data was collated for 20 such sites. Philistine culture represented by large urban sites, such as Ashdod, Ashkelon, Ekron, and Safi/Gath, as well as by small urban sites such as Batash and Qasile, and probably a village—Aphek. Non-Philistine culture in Iron Age is represented here by large urban sites, such as the City of David, Lachish, Megiddo, Rehov, Ta’anach; small urban sites such as Aphek, Batash (Batash is Philistine during part of the Iron Age and Judahite in other, we refer to the appropriate strata as needed), Beth Shean, and Be’er Sheva; and rural sites such as Afula, Arad, Ifshar, Kedesh, Rosh Zayit, and Shiloh^36^.

In addition, plant remains were retrieved from various contexts in all periods. The standard of research and data publication varies somewhat between teams, as does the date of investigation and focus of each excavation. The resulting database includes remains of seeds, fruits and flowers, preserved in silos, sealed vessels, cooking places, floors, street and temples (Supplementary Information Tables S1). Bronze Age strata were represented by more than 570,000 recognized plant remains, while Iron Age strata were represented by more than 430,000 remains. Hence, the compiled database comprises material from a broad geographic range and incorporates data from thousands of recognized plant remains, from numerous sites (>10) within each compared time period, and from various intra-site contexts. The size, the broad chronological time span (Bronze Age period is almost four times longer than the early and late Iron Ages combined), and broad geographic extent of the database, enabled us to investigate the full spectrum of plants accompanying local human activities and to test the diversity between the periods and cultures.

### Data collection for floral lists

Floral list (FL thereafter) for the Bronze Age (n species = 178) was collated for 18 different archaeological sites, which represent a wide range of settlements: Afula, Aphek, Arad, Ashdod, Batash, Beth Shean, Ekron, Jericho, Ifshar, Lachish, Manahat, Megiddo, Nami, Qasile, Shiloh, Shiqmim, Ta’anach, and Safi/Gath^36^. Combining the FLs for the whole Bronze Age facilitated inclusion of as much variation as possible in plant species associated with human activities (synanthropes) from different ecological habitats. Also, combining the FLs for the entire Bronze Age and for areas beyond the geographic range of Philistine settlements enabled us to more precisely pinpoint changes in biodiversity within Israel which were related to shifts in culture, human populations and agrarian activities in the Iron Age.

The Iron Age FLs (n species = 271) was collated for 20 such sites. Philistine culture represented by Aphek, Ashdod, Ashkelon, Batash, Ekron, Qasile, and Safi/Gath. Non-Philistine culture in Iron Age is represented by Afula, Aphek, Arad, Batash (Batash is Philistine during part of the Iron Age and Judahite in other, we refer to the appropriate strata as needed), Be’er Sheva, Beth Shean, City of David, Ifshar, Kedesh, Lachish, Megiddo, Rehov, Rosh Zayit, Shiloh, and Ta’anach^36^.

The FLs included determinations to the genus level and below (Supplementary Information Table S2). Determinations to the *cf.* specific level were treated as species. For each species we validated its taxonomic status to reduce nomenclature bias^39^, and the level of synanthropy^21^. Obligate natural species were classified as ‘species not adapted to grow in synanthropic habitats’, all other species were classified as species adapted to synanthropic habitats (including those classified as mostly natural, also synanthropic; approximately synanthropic as natural; mostly synanthropic, also natural; obligate synanthropic). Cultivated species were classified as synanthropes. Taxa were characterized in FLs by their presence/absence.

Each taxon was categorized according to: (i) its chronological affinity (Bronze Age, early Iron Age, late Iron Age) and (ii) its affinity with an Iron Age Philistine or non-Philistine community (i.e., Canaanites, Israelites, Judahites, and Phoenicians), based on cultural attributes of the associated archaeological assemblages.

### Sites identification as Philistine or non-Philistine settlements

Iron Age sites with archaeobotanical material were identified as Philistine settlements in accordance with archaeological analysis of material culture. Hence, the Philistine sites are: Qasile^40^,^41^, Ekron^42^, Safi/Gath^43^, and Ashkelon^44^. In case of cultural changes within the Iron Age, as in Aphek and Batash, strata with Philistine culture were analyzed separately from the non-Philistine strata of the site. Hence, non-Philistine Aphek was identified with Stratum X11 and Stratum X8, while Philistine Aphek^16^ was identified with Strata X10 and X9. Philistine Batash was identified with Stratum V and then at the beginning of Stratum II, while other Iron Age strata (Strata IV, III), and the most part of Stratum II are Judahite/Israelite^45^.

### History of species in the archaeobotanical record

We analyzed the possible source-regions for the taxa investigated based on published sources for the Bronze (152 sites) and Iron Ages (52 sites) in the Eastern Mediterranean–southwest Asia, including Greece, Turkey, Western Iran, Iraq, Syria, Lebanon, Israel, Jordan, and Northern Egypt^19^,^20^,^21^,^24^,^29^.

### The Method of floral list (FL) comparison

We applied the method of FL comparison to measure the diversity between the periods in terms of taxa and ecology of species. The method of floral list (FL) comparison is based on species present/absence, where presence of a species shared between the FLs marks continuity in environmental conditions. In biogeography and palaeobotany, the method is widely used to distinguish phytogeographical regions and investigate the history of local vegetation^46^,^47^. When applied to archaeobotanical data, the method may elucidate type and intensity of the anthropogenic links and cultural changes^48^,^49^,^50^. Here we used the method to characterize species unique to each time period, and also those which continuously accompanied people at an archaeological site, before, during and after the advent of Philistine culture. Applying the method of FL comparison in archaeology requires accurate dating of the archaeobotanical material to be used for the region being investigated.

### Statistics

We used Rarefaction test to test the possible relation of species ubiquity and sample size (Supplementary Information SI 3). Then, we used Coefficient of Community^13^, CC, to quantify the floristic similarity of the FLs, stressing the amount of taxa shared by them. Dissimilarity of floral lists, D_S_, was calculated using Jaccard similarity coefficient^14^, S_J_, taking into account the amount of the taxa unique to each FL.

CC = 2 x (number of shared taxa)/(SUM of number of taxa in both FL);

the CC varies from zero (no shared taxa) to 1 (same list of taxa in both samples).

D_S_ = 1- S_J_, which is equal to number of shared taxa/(number of shared taxa + number of unique taxa to FL1 + number of unique taxa to FL 2);

the Ds varies from zero (all taxa are shared) to 1 (no shared taxa).

## Additional Information

**How to cite this article**: Frumin, S. *et al.* Studying Ancient Anthropogenic Impacts on Current Floral Biodiversity in the Southern Levant as reflected by the Philistine Migration. *Sci. Rep.* **5**, 13308; doi: 10.1038/srep13308 (2015).

## Supplementary Material

Supplementary Information

## Figures and Tables

**Figure 1:**
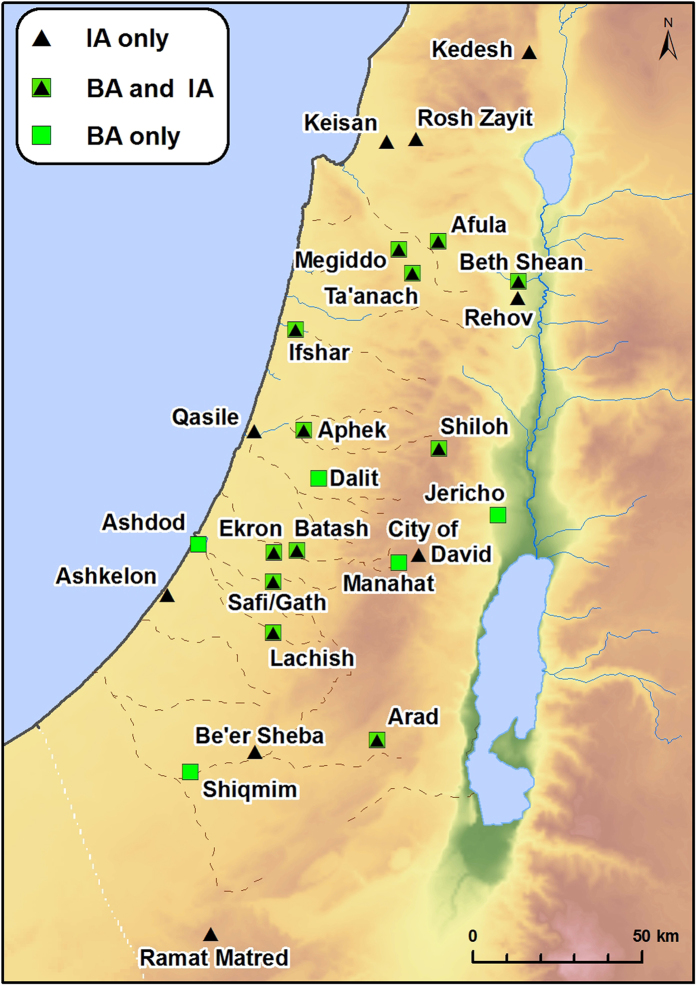
Bronze and Iron Age Archaeobotanical sites in Israel that served as data sources. Green squares denote Bronze Age sites, black triangles denote Iron Age sites, green squares with black triangle inside denote sites with both periods. Map produced by M. Frumin using ArcGIS for Desktop (ArcMap 10.1), ESRI.

**Table 1:** List of new genera and synanthropic species which appeared in the Iron Age (IA) in the Southern Levant.

**Time**	**Philistine -new synanthropic spp.**	**Non-Philistine’- new synanthropic spp.**
Late IA	*Ficus sycamorus*	*Hypericum triquetrifolium*
	*Glaucium corniculatum*	*Persica vulgaris*
	*Malva nicaeensis*	*Prunus armeniaca*
	*Papaver somniferum*	
	*Salsola kali*	
	*Spergula fallax*	
Early IA	*Chenopodium murale/vulvaria*	*Celtis australis*
	*Cichorium endivia*	*Chenopodium murale/vulvaria*
	*Cichorium endivia subsp. divaricatum*	*Citrullus lanatus*
	*Cuminum cyminum*	*Hordeum hexastichium*
	*Echinochloa colonum*	*Ridolfia segetum*
	*Eragrostis barrelieri/pilosa*	
	*Eragrostis pilosa*	
	*Heliotropium europaeum*	
	*Portulaca oleracea*	
	*Trigonella hierosolymitana*	

Data are divided between the early Iron Age, and between Philistine and non-Philistine sites. Plant species names are given in alphabetical order.
