# Supplementary material for: Studying Ancient Anthropogenic Impacts on Current Floral Biodiversity in the Southern Levant as reflected by the Philistine Migration
Source: Sci Rep. 2015 Aug 25;5:13308. doi: 10.1038/srep13308 (PMC4642518; doi:10.1038/srep13308)
Supplement: Supplementary Information [file srep13308-s1.pdf]

Supplementary Information

Studying Ancient Anthropogenic Impacts on Current Floral Biodiversity in the Southern Levant as reflected by the Philistine Migration

Suembikya Frumin[1], Aren M. Maeir[1], Liora Kolska Horwitz[2] & Ehud Weiss[1]

Table S1: Structure of archaeobotanical data for Bronze and Iron Age Sites.

	Bronze Age			Iron Age			
Site Name	Contexts	NN Taxa	NN Plant Remains	Culture	Context	NN Taxa	NN Plant Remains
Afula	Pit, 3 other loci	9	no data	nonPhil	Storage jars	6	41
Aphek	Halls in building, governor palace, street, winepress	81	29180	Phil	Building, dump pits, threshing floor	110	3620
				nonPhil	Settlement, 5 pits, silos, intact jars with grains	50	21398
Arad	Various strata within building - kitchen-storeroom, bins, pithoi, jars	22	60 kg	nonPhil	Various loci from occupation layers	5	>61
Ashdod	2 loci from occupation levels and installation, refuse dump by installation	9	713	0		0	
Ashkelon		0		Phil	Adminstrative center, counting house, shops, streets, silo, warehouse magazines	178	19,543
Be`er Sheba		0		nonPhil	Various loci from occupation Layers	5	ca 30
Beth Shean	Occupation strata, 13 L	53	36768	nonPhil	Building, destruction debris on floor	33	150559

City of David		0		nonPhil	Occupation Layers	12	no data
Dalit	Domestic area	1	no data	0		0	
Ekron	Destruction debris, vessel, storage jar	51	924	Phil	Room floor, pit, fill, fireplaces	91	6940
Rosh Zayit		0		nonPhil	Jars in storeroom	40	184440
Ifshar	Grain` storeroom, food prep. area, courtyard	11	32106	nonPhil	Storage pit near house, 2 other storage pits	15	847
Jericho	silo, 7 tombs, room, pot, 3 shafts, etc.	56	ND	0		0	
Kedesh		0		nonPhil	Pit sealed under floor	1	2
Keisan		0		nonPhil	Silo	9	5235
Lachish	ND	12	167 cm^3	nonPhil	ND	10	300 cm3
Manahat	Sealed contexts on floor, fireplaces and around them	17	411	0		0	
Megiddo	Accumulation on floors, pits, bone accumulation in the temple compound, burials, refuse deposit, plaster floor	28	816	nonPhil	Floors, pits, various installations, buildings. floor, destruction layer, large storage jar	23	348
Qasile		0		Phil	Destruction debris under temple, and adjacent 2 buildings	16	13303
Ramat Matred		0		nonPhil	Occupation Layers	1	1
Safi/Gath	Accumulation floor in building, room	15	249	Phil	Destruction debris, room floor	34	1003
Shiloh	ND, 8 loci	21	357	nonPhil	Silo, 2 loci out of 74 loci	33	22952
Shiqmim	22 samples - lower village building phase	5	22	0		0	
Ta`anach	ND, 7 loci	19	ND	nonPhil	ND, 6 loci	9	ND

Batash	Building, sealed jar, etc at least 3 loci	55	220761	nonPhil	ND, 10 loci	1	ND
				Phil	ND, 9 loci	1	ND
Rehov		0		nonPhil	9 different loci	1	200

Each site is characterized by time period (Bronze Age and Iron Age), cultural affinity of Iron Age Strata (Philistine or non-Philistine), context contained remains of seeds and fruits, number of taxa recognized, and number of plant remains analyzed. Phil – Philistine Strata; nonPhil – other than Philistine Strata within the Iron Age. ND – no data in publication.

Table S2: The archaeobotanical record of Southern Levant in Bronze and Iron Ages. Data based on studies of remnants of flowers, fruits, and seeds. Species ecology is denoted as cult = cultivated species; w/w = wild and/ or weed species. Iron Age Aphek contains both Philistine (Phil) and non-Philistine (nonPhil) strata.

List of Taxa	Ecology	Bronze Age	Iron Age Phil	Iron Age nonPhil
Adonis sp.		Aphek[1, 2]<br>Manahat[3]<br>Safi/Gath[2, 4]	Aphek[1, 2]<br>Ashkelon[2, 5]<br>Safi/Gath[2, 4]	-
Adonis dentata	w/w	Shiqmim[6]	-	-
Adonis dentata/microcarpa	w/w	-	Ashkelon[5]	-
Aegilops geniculata/peregrina	w/w	-	-	Beth Shean [7]
Aegilops sp.	w/w	Shiqmim[6]	Ashkelon[5]	-
Aira elengatissima	w/w	-	Safi/Gath[2, 4]	-
Alkanna strigosa	w/w	-	-	Beth Shean [7]
Amaranthus sp.	w/w	-	-	Megiddo [8]
Ammi majus	w/w	-	Aphek[1, 2]	-
Amygdalus communis	w/w	Ta'anach[9]	Ashkelon[10, 11, 5]	City of David [12]
Anagallis arvensis/foemina	w/w	-	Ashkelon[2]	-
Anagallis arvensis	w/w	Aphek[1, 2]	Aphek[1, 2]<br>Ashkelon [2, 5]<br>Ekron[2]<br>Safi/Gath[2, 4]	Aphek[2]<br>Beth Shean[7]
Anagallis sp.	w/w	Megiddo[8]	-	Megiddo[8]
Androcymbium palaestinum	w/w	-	Ashkelon[5]	-
Anthemis bornmuelleri	w/w	-	Ekron[2]	-
Anthemis cotula/parvifolia	w/w	-	Ashkelon[5]	-
Anthemis palestina	w/w	Batash[13]	Ashkelon[2]	-
Anthemis pseudocotula	w/w	-	Ashkelon[2, 10, 11, 5]<br>Ekron[2]	-
Anthemis tinctoria	w/w	Beth Shean[14]	-	-
Apium graveolens	w/w	Beth Shean[14]	-	-
Apium nodiflorum	w/w	-	Aphek[1, 2]	Aphek[1, 2]
Arnebia decumbens	w/w	Shiqmim[6]	Ashkelon[10, 11, 5]	-
Arrhenatherum palaestinum	w/w	-	Ekron[2]	-

Arundo sp.		-	Ekron[2]	-
Asperula arvensis/Galium tricornutum	w/w	Batash[13]	-	-
Asperula sp.		Batash[13]	-	-
Asphodelus tenuifolius	w/w	-	Ashkelon[2]	-
Astragalus asterias	w/w	Aphek[1, 2]	Aphek[1, 2]<br>Ashkelon[2]	Aphek[2]
Astragalus hamosus	w/w	-	Aphek[1, 2]<br>Ekron[2]	-
Astragalus macrocarpus/aleppicus	w/w	-	Ekron[2]	-
Astragalus sp.		Aphek[1, 2]<br>Ifshar[15, 16]<br>Safi/Gath[2, 4]	Aphek[1, 2]<br>Ashkelon[5]<br>Ekron[2]<br>Safi/Gath[2, 4]	-
Avena barbata	w/w	Aphek[1, 2]<br>Batash[13]	Aphek[1, 2]<br>Ashkelon[2, 5]	-
Avena barbata/sterilis	w/w	Aphek[1, 2]	Aphek [1, 2]	Aphek[1, 2]
Avena sterilis	w/w	Aphek[1, 2]<br>Batash[13]<br>Ekron[2]	-	Aphek[1, 2]
Avena sp.		Afula[17]<br>Batash[13]<br>Beth Shean[14]<br>Ifshar[15, 16]<br>Jericho[18]<br>Manahat[3]<br>Shiqmim[6]	Ekron[2]	Rosh Zayit[19]<br>Shiloh[20]
Bellevalia eigii/flexuosa/warburgii	w/w	-	Ashkelon[5]	-
Bellevalia flexuosa	w/w	Aphek[1, 2]	Aphek[1, 2]<br>Ashkelon[2, 5]<br>Ekron[2]	Rosh Zayit[19]
Bellevalia sp. and *Bellevalia/Muscari*		Aphek[2]<br>Batash[13]	Ashkelon[2]<br>Ekron[2]	Aphek[1, 2]<br>Beth Shean[7]<br>Shiloh[20]
Beta vulgaris	w/w	Aphek[1, 2]	Aphek[1, 2]<br>Ashkelon[2, 5]	-
Brachypodium distachyon	w/w	Aphek[1, 2]<br>Ekron[2]	Aphek[1, 2]<br>Ashkelon[2,5]<br>Ekron[2]	Aphek[1, 2]<br>Beth Shean[7]<br>Rosh Zayit[19]<br>Shiloh[20]
Brassica nigra	w/w	Ekron[2]	Aphek[1, 2]	-
Bromus alopecuros	w/w	Aphek[1, 2]	-	-
Bromus alopecurus/lanceolatus	w/w	-	-	Rosh Zayit[19]
Bromus diandrus	w/w	-	Aphek[1, 2]	-
Bromus tigridis/pseudobrachystachys		-	Aphek[1, 2]	-
Bromus sp.		Aphek[1, 2]<br>Arad[21]<br>Batash[13]<br>Jericho[18]	Aphek[1]<br>Ashkelon[2, 5]<br>Ekron[2]	Megiddo[8]
Buglossoides arvensis	w/w	-	Ashkelon[2, 10, 11, 5]<br>Ekron[2]	Beth Shean[7]
Buglossoides tenuiflora	w/w	Beth Shean[14]	Ashkelon[2, 5]<br>Ekron[2]	-
Bupleurum lancifolium	w/w	Beth Shean[14]<br>Ifshar[15, 16]	Ashkelon[5]	Beth Shean[7]<br>Rosh Zayit[19]<br>Shiloh[13]

Bupleurum subovatum	w/w	Aphek[1, 2]<br>Batash[13]<br>Ekron[2]<br>Safi/Gath[2, 4]	Aphek[1, 2]<br>Ashkelon[2, 5]<br>Ekron[2]<br>Safi/Gath[2, 4]	Aphek[1, 2]
Calendula arvensis	w/w	Beth Shean[14]	-	-
Calendula sp.	w/w	Jericho[18]	-	Beth Shean[7]
Capparis spinosa	w/w	Beth Shean[14]<br>Ta'anach[9]	Aphek[1, 2, 5] Ashkelon[2]	Ta'anach[9]
Carex divisa	w/w	-	Ekron[2]	-
Carex hallerana	w/w	-	Ekron[2]	-
Carthamus sp.		-	Ashkelon[2, 5]	-
Catabrosa aquatica	w/w	-	Ekron[2]	-
Celtis australis	cult	-	-	Rehov[22]
Centaurea crocodylium	w/w	-	-	Shiloh[20]
Centaurea iberica	w/w	Aphek[1, 2]	-	-
Centaurea iberica/hyalolepis	w/w	Batash[13]	-	-
Centaurea pallescens	w/w	-	Ashkelon[2]<br>Ekron[2]	-
Centaurea procurrens	w/w	-	Aphek[1, 2]	-
Centaurea procurrens/glomerata	w/w	-	Aphek[2]	-
Centaurea verutum	w/w	Batash[13]	-	-
Centaurea sp.	w/w	Jericho[18]	-	-
Cephalaria joppensis	w/w	Batash[13]	Ashkelon[2,5]	Keisan[23]<br>Rosh Zayit[19]
Cephalaris joppensis/syriaca	w/w	Ekron[2]	Ekron[2]	-
Cephalaria syriaca	w/w	Batash[13]<br>Ifshar[15, 16]	Aphek[1, 2]	Afula[24]<br>Aphek[1, 2]<br>Beth Shean[25]<br>Keisan[23]<br>Shiloh[20]
Cephalaria sp.	w/w	-	Ashkelon[2, 5]<br>Safi/Gath[2, 4]	-
Ceratonia siliqua	cult	-	Ashkelon[10, 11,5]	-
Chenopodium murale	w/w	Aphek[1, 2]<br>Beth Shean[14]<br>Ekron[2]	Aphek[1, 2]<br>Ashkelon[2, 10, 11, 5]<br>Ekron[2]	Rosh Zayit[19]
Chenopodium murale/vulvaria	w/w	Aphek[1]	Aphek[1, 2]	Aphek[2]
Chrozophora sp.	w/w	Megiddo[8]	Aphek[1, 2]<br>Ekron[2]	-
Chrysanthemum coronarium	w/w	Aphek[1, 2]<br>Beth Shean[14]	Aphek[1, 2]<br>Ashkelon[2, 5]<br>Ekron[2]<br>Safi/Gath[2, 4]	Aphek[1, 2]
Cicer arietinum	cult	Arad[21]<br>Beth Shean[14]<br>Ifshar[15, 16]<br>Jericho[18]<br>Lachish[26]	Ashkelon[10, 11, 5]	Afula[24]<br>Lachish[26]
Cicer pinnatifidum	w/w	Afula[17]	Ashkelon[2, 5]	-
Cichorium endivia	w/w	-	Aphek[1]<br>Ekron[2]	-
Cichorium endivia subsp. *divaricatum*	w/w	-	Aphek[2]	-
Cichorium pumilum	w/w	-	Ashkelon[5]	-

Citrullus colocynthis	w/w	-	-	Arad[27]
Cladium mariscus	w/w	-	Ashkelon[10, 11, 5]	-
Conium maculatum	w/w	Aphek[1, 2]	-	-
Consolida incana	w/w	-	-	Rosh Zayit[19]
Convolvulus arvensis	w/w	Batash[13]	-	Rosh Zayit[19]
Convolvulus oleifolius	w/w	-	Aphek[1, 2]	-
Convolvulus secundus	w/w	-	Ashkelon[2, 5]	Aphek[1, 2]
Convolvulus sp.	w/w	Ekron[2]	Ashkelon[2]	-
Coriandrum sativum	w/w	-	Ashkelon[2]<br>Ekron[2]	-
Coronilla cretica	w/w	-	-	Rosh Zayit[19]
Coronilla repanda	w/w	Ekron[2]	-	-
Coronilla repanda/scorpioides	w/w	-	-	Shiloh[20]
Coronilla scorpioides	w/w	Aphek[1, 2]	Aphek[1, 2]<br>Ekron[2]	Aphek[1, 2]
Coronilla sp.	w/w	Ekron[2]	Ekron[2]<br>Safi/Gath[2, 4]	-
Crataegus sp.		Lachish[26]	-	-
Crepis aspera	w/w	-	-	Rosh Zayit[19]
Crucianella sp.		-	Ashkelon[5]	-
Cuminum cyminum	cult	-	Aphek[1, 2]	-
Cutandia maritima	w/w	-	Ashkelon[2]	-
Cynodon dactylon	w/w	-	Ashkelon[5]	Rosh Zayit[19]
Dactylis glomerata	w/w	Aphek[1, 2]<br>Ekron[2]	-	-
Daucus broteri	w/w	Batash[13]	Aphek[1, 2]	-
Daucus carota subsp. *maximus*	w/w	-	Ashkelon[2, 5]<br>Aphek[1, 2]	-
Echinochloa colonum	w/w	-	Ashkelon[2]	-
Echiochilon fruticosum	w/w	-	Ashkelon[2, 10, 11, 5]	-
Echium angustifolium/judaeum		Safi/Gath[2, 4]	Ashkelon[2]<br>Ekron[2]<br>Safi/Gath[2, 4]	-
Echium judaeum	w/w	Jericho[18]<br>Megiddo [8]<br>Ta'anach[9]	Ashkelon[5]	Ta'anach[9]
Eleocharis palustris	w/w	-	Ashkelon[2]	-
Emex spinosa	w/w	Aphek[1, 2]	Ashkelon[2, 5]	-
Eragrostis barrelieri/pilosa	w/w	-	Ashkelon[2]	-
Eragrostis pilosa	w/w	-	Ekron[2]	-
Erodium botrys	w/w	-	Ekron[2]	-
Erodium moschatum	w/w	-	Ashkelon[2]<br>Aphek[1, 2]	-
Erucaria hispanica	w/w	Batash[13]	-	-
Erucaria microcarpa	w/w	-	Ashkelon[10, 11, 5]	-
Euphorbia helioscopia	w/w	-	Aphek[1, 2]<br>Qasile[28]	-
Euphorbia peplus	w/w	Aphek[1, 2]	-	-
Euphorbia valerianifolia	w/w	Batash[13]	Ashkelon[2]	-
Euphorbia sp.		-	Aphek[1, 5]<br>Ashkelon[2, 5]	-

Ficus carica	cult	Aphek[1, 2]<br>Batash[13]<br>Beth Shean[14]<br>Ekron[2]<br>Ifshar[29]<br>Jericho[18]<br>Megiddo[8]<br>Safi/Gath[2, 4]<br>Shiloh[20]	Aphek[1, 2]<br>Ashkelon[2, 10, 11, 5]<br>Ekron[2]<br>Qasile[28]<br>Safi/Gath[2, 4]	Aphek[1, 2]<br>Megiddo[8]<br>Rosh Zayit[19]<br>Shiloh[20]
Ficus sycomorus	cult	-	Ashkelon[10, 11, 5]	-
Fumaria parviflora	w/w	-	Ashkelon[5]	Beth Shean[7]
Fumaria (Sect. Fumaria)		Aphek[1, 2]<br>Jericho[18]	Aphek[1, 2]<br>Ashkelon[2]	Aphek[1, 2]
Galium aparine	w/w	-	Ashkelon[2, 5]	-
Galium tricornutum	w/w	Beth Shean[14]<br>Manahat[3]<br>Shiloh[20]	Ashkelon[5]	Afula[24]<br>Beth Shean[7]<br>Rosh Zayit[19]<br>Shiloh[20]
Galium sp.		Aphek[2]<br>Batash[13]<br>Ekron[2]<br>Ifshar[15, 29, 16]<br>Jericho[18]	Aphek[1, 2]<br>Ashkelon[2]<br>Ekron[2]	Aphek[1, 2]<br>Ifshar[15]
Gastridium ventricosum	w/w	-	Ekron[2]	-
Geranium sp.		Ekron[2]	-	-
Geropogon hybridus	w/w	Batash[13]	-	-
Glaucium corniculatum	w/w	-	Ashkelon[2]	-
Gynandriris sisyrinchium	w/w	-	Aphek[1, 2]<br>Ashkelon[5]	Beth Shean[7]
Gypsophyla sp.		Megiddo[8]	-	Megiddo[8]
Helianthemum stipulatum	w/w	-	Ashkelon[10, 11, 5]	-
Heliotropium europaeum	w/w	-	Aphek[1, 2]<br>Safi/Gath[2, 4]	-
Heliotropium sp.		Aphek[1, 2]	Ashkelon[2]<br>Ekron[2]	Shiloh[20]
Hippocrepis areolata/unisiliquosa	w/w	-	Ashkelon[5]	-
Hippocrepis unisiliquosa	w/w	-	Aphek[1, 2]	-
Hippocerpis sp.		-	-	Shiloh[20]
Hordeum bulbosum	w/w	Aphek[1, 2]	-	-
Hordeum distichon	cult	Arad[21]<br>Ifshar[15, 16]<br>Jericho[18]<br>Manahat[3]<br>Shiloh[20]	Aphek[1, 2]<br>Ashkelon[10, 11]<br>Qasile[28]	Beth Shean[7, 25]<br>Ifshar[15]<br>Rosh Zayit[19]<br>Shiloh[20]
Hordeum hexastichium	cult	-	-	Beth Shean[7]
Hordeum glaucum	w/w	Aphek[1, 2]	Ashkelon[2, 5]<br>Ekron[2]<br>Safi/Gath[2, 4]	Aphek[1, 2]<br>Beth Shean[7]
Hordeum marinum	w/w	-	Ashkelon[2, 5]	-
Hordeum vulgare s.l. (*Hordeum sativum* and *Hordeum vulgare*)	cult	Afula[17]<br>Aphek[1, 2]<br>Arad[21]<br>Batash[13]<br>Beth Shean[14]<br>Ekron[2]	Aphek[1, 2]<br>Ashkelon[2, 5]<br>Ekron[2]<br>Qasile[28]<br>Safi/Gath[2, 4]	Aphek[1, 2]<br>Beth Shean[25]<br>Keisan[23]<br>Megiddo[8]<br>Ramat Matred[31]<br>Rosh Zayit[19]

		Ifshar[15, 29, 16]<br>Jericho[18]<br>Lachish[26]<br>Megiddo[8]<br>Safi/Gath[2, 4]<br>Shiqmim[6]		Shiloh[20]
Hordeum spontaneum	w/w	-	Ashkelon[2, 5]	Rosh Zayit[19]
Hordeum sp.	w/w	Afula[17]<br>Ashdod [32]<br>Ifshar[15]<br>Jericho[18]<br>Ta'anach[9]	Ekron[2]	Arad[27]<br>Be'er Sheba [27]<br>City of David[12]<br>Megiddo[8]<br>Ramat Matred[31]
Hymenocarpos circinnatus	w/w	Aphek[1, 2]	Aphek[1, 2]<br>Ashkelon[5]<br>Ekron[2]	-
Hyoscyamus albus	w/w	-	Ashkelon[2]	-
Hyoscyamus aureus	w/w	-	Ashkelon[2]	-
Hypericum triquetrifolium	w/w	-	-	Rosh Zayit[19]
Juglans regia	cult	-	-	City of David[12]
Juncus acutus	w/w	-	Ashkelon[10, 11, 5]	-
Kickxia spuria	w/w	-	Ashkelon[2]	-
Knautia integrifolia	w/w	Megiddo[8]	-	Megiddo[8]
Lathyrus clymenum	w/w	Nami[33, 34]	-	-
Lathyrus hierosolymitanus	w/w	-	Aphek[1, 2]<br>Ekron[2]	-
Lathyrus marmoratus	w/w	-	Ekron[2]	-
Lathyrus sativus	cult	Ekron[2]<br>Lachish[26]	Ashkelon[2, 5]	-
Lathyrus sativus/cicera	cult	Batash[13]	Ashkelon[34]<br>Ekron[34]<br>Qasile[28, 34]	-
Lathyrus (Sect. Cicercula)	cult	Ashdod [32]<br>Batash[13]<br>Ekron[2]	Aphek[1, 2]<br>Ashkelon[2, 5]<br>Ekron[2]	Aphek[1, 2]<br>Shiloh[20]
Lathyrus sp.		Ekron[2]<br>Jericho[18]<br>Shiloh[20]	Ashkelon[2, 10, 11, 5]	Rosh Zayit[19]
Laurus nobilis	w/w	-	Ashkelon[10, 11, 5]	-
Lavatera trimestris	w/w	Batash[13]	-	-
Lens culinaris	cult	Aphek[1, 2]<br>Arad[21]<br>Batash[13]<br>Beth Shean[14]<br>Ekron[2]<br>Ifshar[15, 16]<br>Jericho[18]<br>Lachish[26]<br>Manahat[3]<br>Megiddo[8]<br>Shiloh[20]<br>Safi/Gath[2, 4]	Aphek[1, 2, 11]<br>Ashkelon[2, 10, 5]<br>Ekron[2]<br>Qasile[28]<br>Safi/Gath[2, 4]	Aphek[1, 2]<br>Beth Shean[7]<br>Ifshar[15]<br>Megiddo[8]<br>Shiloh[20]
Lens sp.		Aphek[1, 2]<br>Ifshar[29]<br>Jericho[18]<br>Ta'anach[9]	Ekron[2]	Aphek[2]<br>City of David [12]

Leopoldia comosa	w/w	-	-	Aphek[1, 2]
Linaria chalepensis	w/w	-	Ashkelon[10, 11, 5]	-
Linum bienne	w/w	-	Ashkelon[2, 5]	-
Linum bienne/nodiflorum	w/w	-	-	Beth Shean[25]
Linum bienne/strictum	w/w	-	Ekron[2]	-
Linum nodiflorum	w/w	Batash[13]	-	Rosh Zayit[19]
Linum pubescens	w/w	Batash[13]	-	-
Linum usitatissimum	cult	Aphek[1, 2]<br>Arad[21]<br>Beth Shean[14]<br>Jericho[18]	Aphek[1, 2]<br>Ashkelon[2, 5]<br>Ekron[2]<br>Qasile[28]	Aphek[1, 2]<br>Beth Shean[25]<br>Shiloh[20]
Linum sp.		Aphek[2]<br>Beth Shean[14]<br>Ekron[2]<br>Ifshar[15, 29, 16]<br>Jericho[18]	Aphek[1, 2]<br>Ashkelon[2, 5]<br>Ekron[2]	Aphek[1, 2]<br>Ifshar[15]
Lolium rigidum	w/w	Beth Shean[14]	Ashkelon[2, 5]	Rosh Zayit[19]
Lolium temulentum	w/w	Aphek[1, 2]<br>Batash[13]<br>Beth Shean[14]<br>Ekron[2]<br>Ifshar[15, 29, 16]<br>Lachish[26]<br>Megiddo[8]<br>Shiloh[20]<br>Safi/Gath[2, 4]	Aphek[1, 2]<br>Ashkelon[2, 10, 11, 5]<br>Ekron[2]<br>Qasile[28]<br>Safi/Gath[2, 4]	Aphek[1, 2]<br>Beth Shean[7, 25]<br>Ifshar[15]<br>Keisan[23]<br>Lachish[26]<br>Megiddo[8]<br>Rosh Zayit[19]<br>Shiloh[20]
Lolium temulentum/rigidum	w/w	-	-	Beth Shean[7]
Lolium sp.		Arad[21]<br>Beth Shean[14]<br>Jericho[18]<br>Manahat[3]<br>Megiddo [8]	-	Megiddo[8]
Lupinus sp.		-	-	Rosh Zayit[19]
Lycium schweinfurthii	w/w	-	Aphek[1, 2]	-
Lysimachia dubia	w/w	-	Ashkelon[10, 11, 5]	-
Malva nicaeensis	w/w	-	Ashkelon[2]	-
Malva oxyloba/parviflora	w/w	-	Ashkelon [5]	-
Malva parviflora	w/w	Aphek[1, 2]<br>Beth Shean[14]<br>Shiloh[20]<br>Shiqmim[6]	Aphek[1, 2]<br>Ashkelon[2, 5]<br>Safi/Gath[2, 4]	-
Malva sp.		Aphek[1, 2]<br>Arad[21]<br>Beth Shean[14]<br>Ekron[2]<br>Ifshar[15, 29, 16]<br>Jericho[18]<br>Safi/Gath[2, 4]	Aphek[1, 2]<br>Ashkelon[2, 5]<br>Ekron[2]<br>Safi/Gath[2, 4]	Aphek[1, 2]
Medicago doliata	w/w	Ekron[2]	-	-
Medicago littoralis	w/w	-	Aphek[1, 2]	-
Medicago minima	w/w	Beth Shean[14]<br>Megiddo[8]	-	Megiddo[8]
Medicago orbicularis	w/w	Ta'anach[9]	-	-
Medicago polymorpha	w/w	-	Aphek[1, 2]	-

Medicago polymorpha/minima		-	Aphek[1, 2]	-
Medicago scutellata	w/w	Ta'anach[9]	-	-
Medicago tuberculata	w/w	-	Ashkelon[5]	Beth Shean[7]
Medicago sp.		Aphek[1, 2]<br>Batash[13]<br>Ekron[2]<br>Ifshar[15,16, 29]<br>Safi/Gath[2, 4]	Aphek[1, 2]<br>Ashkelon[2, 5]<br>Ekron[2]<br>Safi/Gath[2, 4]	Aphek[1, 2]
Melilotus sp.		Megiddo[8]	-	Megiddo[8]
Mercurialis annua	w/w	Aphek[1, 2]	Aphek[1, 2]<br>Ekron[2]	Aphek[2]
Mesembryanthemum sp.	w/w	-	Ashkelon[5]	-
Moltkiopsis ciliata	w/w	-	Ashkelon[2]	-
Neslia apiculata	w/w	-	-	Rosh Zayit [19]<br>Shiloh[20]
Ochthodium aegyptiacum	w/w	Aphek[1, 2]<br>Ekron[2]	Ashkelon[2, 5]	Aphek[2]<br>Shiloh[20]
Olea europaea	cult	Aphek[1, 2]<br>Arad[21]<br>Batash[13]<br>Beth Shean[14]<br>Ekron[2]<br>Lachish[26]<br>Manahat[3]<br>Megiddo[8]<br>Safi/Gath[2, 4]<br>Shiloh[20]<br>Ta'anach[9]<br>Tel Dalit[35]	Aphek[1, 2]<br>Ashkelon[2, 10, 11, 5]<br>Batash[13]<br>Ekron[2]<br>Safi/Gath[2, 4]	Afula[24]<br>Aphek[1, 2]<br>Arad[27]<br>Batash[13]<br>Be'er Sheba[27]<br>Beth Shean[7]<br>City of David[12]<br>Kedesh[36]<br>Lachish[26]<br>Megiddo[8]<br>Shiloh[20]<br>Ta'anach[9]
Onobrychis caput-galli	w/w	Shiloh[20]	Ashkelon[5]	-
Onobrychis sp.		-	Ashkelon[2]	-
Ononis ornithopodioides/viscosa	w/w	Shiloh[20]	-	-
Ononis serrata	w/w	Beth Shean[14]	-	-
Ononis viscosa ssp. sicula	w/w	-	Aphek[1, 2]	-
Ononis sp.		Aphek[1, 2]<br>Ifshar[15, 16]	Aphek[1, 2]	-
Onopordum sp.	w/w	-	Ashkelon[5]	-
Onosma gigantea	w/w	-	Aphek[1, 2]<br>Ashkelon[2]<br>Ekron[2]	-
Oryzopsis miliacea	w/w	-	-	Shiloh[20]
Panicum sp.		-	Ekron[2]	-
Papaver somniferum	cult	-	Ashkelon[2]	-
Papaver sp.	w/w	Jericho[18]	-	-
Paronychia sp.		Beth Shean[14]	-	-
Phalaris paradoxa	w/w	Aphek[1]<br>Batash [13]<br>Beth Shean[14]<br>Ekron[2]<br>Ifshar[15, 29, 16]<br>Megiddo[8]<br>Shiloh[20]	Aphek[1, 2]<br>Ashkelon[10, 11, 5]	Ifshar[15]<br>Keisan[23]<br>Megiddo[8]<br>Rosh Zayit[19]<br>Shiloh[20]
Phalaris paradoxa/minor	w/w	Afula[17]<br>Beth Shean[14]	-	Beth Shean[7]

Phalaris sp.		Aphek[1, 2]<br>Ekron[2]<br>Safi/Gath[2, 4]	Aphek[1, 2]<br>Ashkelon[2]<br>Ekron[2]<br>Safi/Gath[2, 4]	Aphek[1, 2]
Phleum exaratum/subulatum	w/w	-	Safi/Gath[2]	-
Phleum subulatum and *Phleum* sp.	w/w	-	Aphek[1, 2]<br>Ashkelon[2,5]	-
Phoenix dactylifera	cult	Jericho[18]	-	Arad[27]<br>Be'er Sheba[27]<br>City of David[12]<br>Ramat Matred[31]
Picris altissima	w/w	Batash[13]<br>Beth Shean[14]	-	Rosh Zayit [19]
Picris amalecitana	w/w	-	Ashkelon[2]	-
Picris galilaea	w/w	Batash[13]	Ashkelon[2, 10, 11, 5]	-
Piptatherum holciforme	w/w	-	Ashkelon[2, 5]	-
Piptatherum sp.		-	Aphek[1, 2]	-
Pistacia atlantica	w/w	Arad[21]<br>Batash[13]<br>Beth Shean[14]<br>Jericho[18]<br>Lachish[26]<br>Manahat[3]	Aphek[1, 2]<br>Ekron[2]	-
Pistacia atlantica/palaestina	w/w	-	Ekron[2]	-
Pistacia palaestina	w/w	Ekron[2]<br>Manahat[3]<br>Shiloh[20]	-	Shiloh[20]
Pisum elatius	w/w	-	Aphek[1, 2]	-
Pisum fulvum	w/w	-	Safi/Gath[2, 4]	-
Pisum sativum	cult	Arad[21]<br>Jericho[18]	-	-
Pisum sp.		Ta'anach[9]	-	-
Plantago lagopus	w/w	-	Ashkelon[2]	-
Plantago notata	w/w	-	Ashkelon[5]	-
Plantago ovata	w/w	-	Ekron[2]	-
Plantago sp.		Aphek[1, 2]<br>Ekron[2]<br>Jericho[18]<br>Shiqmim[6]	Ashkelon[2, 5]	Aphek[2]
Poa bulbosa	w/w	-	Ashkelon[5]	-
Poa sp.		-	Ashkelon[2, 5]	-
Polygonum arenastrum	w/w	Aphek[1, 2]	Ashkelon[5]	-
Polygonum sp.		Megiddo[8]	-	Megiddo[8]
Portulaca oleracea	w/w	-	Ashkelon[2,5]	-
Prosopis farcta	w/w	Beth Shean[14]	-	-
Prunus armeniaca	cult	-	-	City of David[12]
Prunus persica	cult	-	-	City of David[12]
Prunus sp.		-	Qasile[28]	-
Punica granatum	cult	Arad[21]<br>Batash[13]<br>Beth Shean[14]<br>Ekron[2]<br>Ifshar[15]	Ashkelon[10, 11]<br>Aphek[1, 2]<br>Ekron[2]<br>Safi/Gath[2, 4]	City of David[12]

		Jericho[18]<br>Shiloh[20]		
Quercus sp.		Arad[21]<br>Ifshar[15, 16]<br>Manahat[3]	Ashkelon[10, 11, 5]	-
Ranunculus arvensis	w/w	Aphek[1, 2]	Aphek[1, 2, 5]	-
Ranunculus marginatus	w/w	Batash[13]	-	-
Ranunculus marginatus var. *scandicinus*	w/w	Aphek[1, 2]<br>Ekron[2]	-	Aphek[1, 2]
Raphanus raphanistrum	w/w	-	Ashkelon[2, 5]	-
Rapistrum rugosum	w/w	Aphek[1, 2]<br>Ekron[2]	Aphek[1, 2]<br>Ashkelon[5]<br>Ekron[2]<br>Safi/Gath[2, 4]	Shiloh[20]
Reboudia pinnata	w/w	-	Ashkelon[2, 5]	-
Reseda orientalis	w/w	-	Ashkelon[2]	-
Reseda sp.		Aphek[1, 2]	-	-
Retama raetam	w/w	-	Ashkelon[5]	-
Rhagadiolus stellatus	w/w	Batash[13]	-	-
Ridolfia segetum	w/w	-	-	Rosh Zayit[19]
Rubus sanguineus	w/w	Shiloh[20]	-	-
Rumex conglomeratus	w/w	Beth Shean[14]	-	-
Rumex pulcher	w/w	Aphek[1, 2]<br>Batash[13]<br>Ekron[2]<br>Megiddo[8]	Aphek[1, 2]<br>Ashkelon[2, 5]<br>Ekron[2]	Aphek[1, 2]<br>Megiddo[8]<br>Rosh Zayit[19]<br>Shiloh[20]
Salsola kali	w/w	-	Ashkelon[2]	-
Schoenus nigricans	w/w	-	Aphek[1, 2]<br>Ashkelon[2, 10, 11, 5]<br>Ekron[2]<br>Safi/Gath[2, 4]	-
Scirpus holoschoenus	w/w	Beth Shean[14]	-	-
Scirpus lacustris	w/w	-	Aphek[1, 2]	-
Scirpus litoralis	w/w	Aphek[1, 2]	Aphek[1, 2]<br>Ashkelon[2]<br>Ekron[2]	-
Scirpus maritimus	w/w	Aphek[1, 2]<br>Beth Shean[14]	Aphek[1, 2]	Aphek[2]
Scirpus maritimus/lacustris	w/w	Aphek[1, 2]	Aphek[1, 2]<br>Ashkelon[2]<br>Ekron[2]	-
Scorpiurus muricatus	w/w	Aphek[1, 2]<br>Ashdod[32]<br>Batash[13]<br>Beth Shean[14]<br>Ekron[2]<br>Lachish[26]<br>Safi/Gath[2, 4]<br>Shiloh[20]	Aphek[1, 2]<br>Ashkelon[2, 5]<br>Ekron[2]<br>Qasile[28]<br>Safi/Gath[2, 4]	Aphek[1, 2]<br>Beth Shean[7]
Scorpiurus sp.		Megiddo[8]	-	-
Securigera securidaca	w/w	-	Aphek[1, 2]<br>Ashkelon[2]	Rosh Zayit[19]
Sherardia arvensis	w/w	-	Ashkelon[5]	-
Silene nocturna	w/w	-	Ashkelon[5]	-

Silene sp.		Ifshar[15, 29, 16]	Ashkelon[2, 5]<br>Ekron[2]	Ifshar[16]
Silybum marianum	w/w	Ashdod[32]	-	-
Sinapis sp.	w/w	Arad[21]	-	-
Sixalix arenaria	w/w	-	Ashkelon[2, 5]	-
Solanum nigrum/villosum	w/w	Beth Shean[14]	Ashkelon[2]	-
Solanum villosum	w/w	Aphek[1, 2]	-	Aphek[2]
Spergula arvensis	w/w	Aphek[1, 2]	Ashkelon [5]	-
Spergula fallax	w/w	-	Ashkelon[2, 5]	-
Stipa arabica	w/w	Beth Shean[14]	-	-
Stipa bromoides	w/w	-	Ashkelon[5]	-
Styrax officinalis	w/w	Manahat[3]	-	-
Suaeda sp.	w/w	Beth Shean[14]	-	-
Tetragonolobus palaestinus	w/w	Ta'anach[9]	-	-
Teucrium capitatum	w/w	-	Ashkelon[5]	-
Thesium humile	w/w	-	Ashkelon[5]	-
Thymelaea hirsuta	w/w	Aphek[1, 2]	Aphek[1, 2]<br>Ashkelon[2, 5]<br>Ekron[2]	Aphek[2]
Thymelaea passerina	w/w	Batash[13]<br>Beth Shean[14]<br>Ekron[2]<br>Megiddo[8]<br>Safi/Gath[2, 4]	Aphek[1, 2]<br>Ashkelon[2, 5]<br>Ekron[2]<br>Safi/Gath[2, 4]	Beth Shean[7]<br>Megiddo[8]<br>Rosh Zayit[19]<br>Shiloh[20]
Torilis sp.		Ekron[2]	-	Rosh Zayit[19]
Trifolium arvense	w/w	Beth Shean[14]	-	-
Trifolium berytheum	w/w	Batash[13]	-	-
Trifolium clusii	w/w	-	Aphek[1, 2]	-
Trifolium spumosum	w/w	-	Aphek[1, 2]	-
Trifolium sp.		Megiddo[8]	Aphek[1, 2]	Beth Shean[7]<br>Megiddo[8]<br>Rosh Zayit[19]
Trigonella arabica	w/w	Shiqmim[6]	-	-
Trigonella foenum-graecum	w/w	Batash[13]<br>Ekron[2]	Ashkelon[5]	Lachish[26]
Trigonella foenum-graecum/berythea	w/w	-	Aphek[1, 2]	-
Trigonella hierosolymitana	w/w	-	Aphek[1, 2]	-
Trigonella sp.	w/w	Aphek[1, 2]<br>Batash[13]	Aphek[1, 2]	-
Triticum/Aegilops		Ashdod [32]	-	-
Triticum aestivum	cult	Arad[21]<br>Jericho[18]<br>Megiddo[8]	-	Megiddo[8]
Triticum compactum	cult	-	-	Lachish[26]
Triticum dicoccum/monococcum	cult	Jericho[18]	-	-
Triticum durum subsp. *horanicum*	cult	-	-	Keisan[23]
Triticum monococcum subsp. *monococcum*	cult	Arad[21]<br>Jericho[37]	-	Lachish[26]
Triticum turgidum subsp. *dicoccoides*	w/w	Arad[21]<br>Jericho[18]	-	-
Triticum turgidum subsp. *dicoccum*	cult	Afula[17]<br>Aphek[1, 2]	Aphek[1, 2]<br>Ashkelon[2, 10, 11, 5]	Aphek[1, 2]<br>Beth Shean[7]

		Arad[21]<br>Batash[13]<br>Beth Shean[14]<br>Ekron[2]<br>Ifshar[15, 29, 16]<br>Jericho[18]<br>Lachish[26]<br>Megiddo[8]<br>Safi/Gath[2, 4]<br>Shiqmim[6]	Ekron[2]<br>Qasile[28]<br>Safi/Gath[2, 4]	Ifshar[15]<br>Keisan[23]<br>Lachish[26]<br>Megiddo[8]<br>Rosh Zayit[19]
Triticum turgidum subsp. *dicoccum/parvicoccum*	cult	Batash [13]	-	-
Triticum turgidum subsp. *parvicoccum*	cult	Afula[17]<br>Aphek[1, 2]<br>Batash[13]<br>Beth Shean[14]<br>Ekron[2]<br>Ifshar[15, 29, 16]<br>Manahat[3]<br>Safi/Gath[2, 4]<br>Shiloh[20]	Aphek[1, 2]<br>Ashkelon[2, 10, 11, 5]<br>Ekron[2]<br>Safi/Gath[2, 4]	Aphek[1, 2]<br>Beth Shean[7, 25]<br>Ifshar[15]<br>Rosh Zayit[19]<br>Shiloh[20]
Triticum sp.		Beth Shean[14]<br>Ta'anach[9]	-	Arad [27]<br>Be'er Sheba[27]<br>City of David[12]<br>Megiddo[8]<br>Ta'anach[9]
Vaccaria hispanica	w/w	Batash[13]	-	Shiloh[20]
Valerianella muricata	w/w	Beth Shean[14]	Ashkelon[2]	Beth Shean[7]
Verbena officinalis	w/w	Megiddo[8]	-	Megiddo[8]
Veronica sp.	w/w	Jericho[18]	-	-
Vicia ervilia	w/w	Aphek[1, 2]<br>Ashdod[32]<br>Batash[13]<br>Ekron[2]<br>Ifshar[15, 29 , 16]<br>Jericho[18]<br>Manahat[3]<br>Megiddo[8]<br>Shiloh[20]	Aphek[1, 2]<br>Ashkelon[2, 10, 11, 5]<br>Ekron[2]<br>Safi/Gath[2, 4]	Afula[24]<br>Aphek[1, 2]<br>Ifshar[15]<br>Keisan[23]<br>Megiddo[8]<br>Shiloh[20]
Vicia faba	cult	Aphek[1, 2]<br>Ashdod[32]<br>Batash[13]<br>Ekron[2]<br>Ifshar[15]<br>Megiddo[8]<br>Safi/Gath[2, 4]	Aphek[1, 2]<br>Ekron[2]<br>Qasile[28]<br>Safi/Gath[2, 4]	Afula[24]<br>Aphek[1, 2]<br>Ifshar[15, 29]<br>Lachish[26]<br>Megiddo[8]<br>Rosh Zayit[19]<br>Shiloh[20]
Vicia galilaea	w/w	Shiloh[20]	-	-
Vicia narbonensis	w/w	-	-	Rosh Zayit[19]
Vicia narbonensis/Pisum	w/w	Batash[13]	Qasile[28]	-
Vicia palaestina	w/w	Ifshar[15, 16]	-	Ifshar[16]
Vicia sativa	cult	Jericho[18]	-	-
Vicia sp.		Aphek[2]<br>Arad[21]<br>Ekron[2]<br>Jericho[18]<br>Lachish[26]<br>Ta'anach[9]	Aphek[2]<br>Ashkelon[2, 5]<br>Ekron[2]	Aphek[2]<br>City of David[12]<br>Lachish[26]

Vigna luteola	w/w	-	Ashkelon[2, 34, 5]	-
Vitex agnus-castus and *Vitex* sp.	w/w	Aphek[1, 2]<br>Ekron[2]	Aphek[1, 2]<br>Ashkelon[2, 5]<br>Ekron[2]	Aphek[1, 2]
Vitis vinifera	cult	Aphek[1, 2]<br>Arad[21]<br>Ashdod [32]<br>Batash[13]<br>Beth Shean[14]<br>Ekron[2]<br>Ifshar[29]<br>Jericho[18]<br>Lachish[26]<br>Manahat[3]<br>Megiddo[8]<br>Safi/Gath[2, 4]<br>Shiloh[20]<br>Ta'anach[9]	Aphek[1, 2]<br>Ashkelon[2, 10, 11, 5]<br>Ekron[2]<br>Qasile[28]<br>Safi/Gath[2, 4]	Aphek[1, 2]<br>City of David[12]<br>Lachish[26]<br>Megiddo[8]<br>Rosh Zayit[19]<br>Shiloh[20]
Total for BA FL: NN of taxa =	178			
NN of species (%) =	115 (65%)			
NN of cult. =	22 (12%)			
NN of w/w =	121 (68%)			
Total for IA FL: NN of taxa =	271			
NN of species =	190 (70%)			
NN of cult. =	29 (11%)			
NN of w/w =	202 (75%)			

1 Kislev, M. E. & Mahler-Slasky, Y. in *Aphek-Antipatris II: The Remains on the Acropolis. Bronze and Iron Age Remains from Areas X, A and G* Vol. 27 *Monograph Series* (eds Gadot, Y. & Yadin, E.) 499-525 (The Institute of Archaeology, Tel-Aviv University, 2009).

2 Mahler-Slasky, Y. *Philistine material culture as reflected by the archaeobotanical remnants from Ashkelon, Ekron, Gath and Aphek.* PhD thesis, Bar-Ilan University, Israel (2004).

3 Kislev, M. E. in *Villages, Terraces and Stone Mounds: Excavations at Manahat* Vol. 3 (eds Edelstein, G. Milevski, I. & Aurant, S.) 113-118 (Israel Antiquities Authority Reports 1998).

4 Mahler-Slasky, Y. & Kislev, M. E. in *Tell es-Safi I: Report on the 1996-2005 Seasons* Vol. ÄAT 69 (ed Maeir, A.) 579-587 (2012).

5 Weiss, E., Kislev, M. E. & Mahler-Slasky, Y. in *Ashkelon 3: The Seventh Century B.C.* (eds Stager, L.E. Master, D.M. & Schloen, J.D.) 591-613 (Eisenbrauns, 2011).

6 Kislev, M. E. in *Shiqmim I, Studies Concerning Chalcolithic Societies in the Northern Negev Desert, Israel (1982-1984)* Vol. 356 (i, ii) (ed Levy, T.E.) 251-279, 549-563 (BAR International Series, 1987).

7 Simchoni, O. & Kislev, M. E. in *Excavations at Tel Beth-Shean 1989-1996* Vol. I (ed Mazar, A.) 679-685 (The Israel Exploration Society and the Institute of Archaeology, HUJ 2006).

8 Borojevic, K. in *Megiddo IV: the 1998-2002 Seasons* Vol. 24 *Monograph Series of the Institute of Archaeology University of Tel-Aviv,* Ch. 27, 519-541 (2006).

9 Liphschitz, N. & Waisel, Y. Dendroarchaeological investigations in Israel (Taanach). *Israel Exploration Journal* **30**, 132-136 (1980).

10 Weiss, E. & Kislev, M. E. in *Ashkelon: A City on the Seashore* (eds Sasson, A. Safrai, Z. & Sagiv, N.) 6-7 (Ashkelon Academic College, 2001).

11 Weiss, E. & Kislev, M. E. Plant remains as indicators for economic activity: a case study from Iron Age Ashkelon. *Journal of Archaeological Science* **31**, 1-13 (2004).
12 Liphschitz, N., Waisel, Y., Fahn, A., Werker, E. & Baruch, U. in *Excavations at the City of David 1978-1985 Directed by Yigal Shiloh. Vol. III. Stratigraphic, Environmental, and Other Reports.* Qedem 33 (eds de Groot, A. , & Ariel, D.T.) 105-121 (Institute of Archaeology, The Hebrew University of Jerusalem, 1992).
13 Kislev, M. E., Melamed, Y. & Langsam, Y. in *Timnah (Tel Batash) III: The Finds from the Second Millennium BCE* Vol. 45 *Qedem* (eds Panitz-Cohen, N. & Mazar, A.) 295-311 (The Hebrew University, 2006).
14 Simchoni, O., Kislev, M. E. & Melamed, Y. in *Excavations at Tel Bet-Shean 1989-1996. Vol. II, the Middle and Late Bronze Age strata in Area R* (eds Mazar, A. & Mullins, R.A.) 702-715 (The Israel Exploration Society and The Institute of Archaeology, The Hebrew University, 2007).
15 Chernoff, M. C. *The Archaeobotanical Material from Tel el Ifshar*, PhD thesis, Brandeis University (1988).
16 Chernoff, M. C. Natural Resource Use in an Ancient Near East Farming Community. *Agricultural History* **66**, 213-220 (1992).
17 Melamed, Y. Dry and charred grains from 'Afula - a taphonomic approach. *Atiqot* **30**, 69-70 (1996).
18 Hopf, M. in *Excavations at Jericho* (eds Kenyon, K. M. & Holland, T. A.) 576-621 (British School of Archaeology in Jerusalem, 1983).
19 Kislev, M. E. & Melamed, Y. in *Horbat Rosh Zayit: an Iron Age storage fort and village* Vol. 8 (eds Gal, Z. & Alexandre, Y.) 206-220 (Israel Antiquities Authority Reports 2000).
20 Kislev, M. E. in *Shiloh: The Archaeology of a Biblical Site* (eds Finkelstein, I. Bunimovich, S. & Lederman, Z.) 354-361 (Institute of Archaeology of Tel Aviv University, 1993).
21 Hopf, M. in *Early Arad I, the Chalcolithic Settlement and Early Bronze City* (ed Amiran, R.) 64-82 (The Israel Exploration Society, 1978).
22 Simchoni, O. & Kislev, M. E. Early finds of *Celtis australis* in southern Levant. *Vegetation History and Archaeobotany*. **20**, 267-271 (2011).
23 Kislev, M. E. in *Tell Keisan (1971-1976): Une cité phénicienne en Galilée* (eds Briend, J. & Humbert, J.-B.) 361-379, pl. 139-140 (Orbis Biblicus et Orientalius, Series Archaeologica 1, 1980).
24 Zaitschek, D. V. Remains of cultivated plants from Afula. *Atiqot* **1**, 71-74 (1995).
25 Kislev, M. E., Simchoni, O., Melamed, Y. & Maoz, L. Flax seed production: evidence from the early Iron Age site of Tel Beth-Shean, Israel and from written sources. *Vegetation History and Archaeobotany*. **20**, 569-584 (2011).
26 Helbaek, H. in *Lachish (Tell ed-Duweir) IV: The Bronze Age* (ed Olga Tufnell) 309-317 (Oxford University Press, 1958).
27 Liphschitz, N. & Waisel, Y. Dendroarchaeological investigations in Israel (Tel Beersheba and Arad in the Northern and Eastern Negev. *Israel Exploration Journal* **23**, 30-36 (1973).
28 Kislev, M. E. & Hopf, M. Food remains from Tell Qasile: with special reference to *Lathyrus sativus/ cicera*. 140-147 (The Institute of Archaeology. The Hebrew University, Jerusalem, 1985).
29 Chernoff, M. C. & Paley, S. M. Dynamics of Cereal Production at Tell el Ifshar during the Middle Bronze Age. *Journal of Field Archaeology* **25**, 397-416 (1998).
30 Kislev, M. E. in *Jerusalem and Eretz Israel* (eds Schwarz, J. Amar, Z. & Ziffer, I.) 23-30 (Eretz Israel Museum, 2000).
31 Hopf, M. & Zachariae, G. Determination of Botanical and Zoological Remains from Ramat Matred and Arad. *Israel Exploration Journal* **21**, 60-64 (1971).
32 Melamed, Y. Botanical remains from the excavation on the southern beach of Ashdod. *Atiqot* **74**, 127-131 (2013).
33 Kislev, M. E., Artzy, M. & Marcus, E. Import of an Aegean food plant to a Middle Bronze IIA coastal site in Israel. *Levant* **25**, 145-154 (1993).
34 Mahler-Slasky, Y. & Kislev, M. E. *Lathyrus* consumption in late Bronze and iron age sites in Israel: an Aegean affinity. *Journal of Archaeological Science* **37**, 2477-2485, doi:DOI: 10.1016/j.jas.2010.05.008 (2010).
35 Liphschitz, N. in *Excavations at Tel Dalit: an Early Bronze Age Walled Town in Central Israel* (ed Gophna, R.) 186-192 (1996).
36 Borojevic, K. Interpreting, dating, and reevaluating the botanical assemblage from tell Kedesh: a case study of historical contamination. *Journal of Archaeological Science* **38**, 829-842, doi:http://dx.doi.org/10.1016/j.jas.2010.11.005 (2011).
37 Hopf, M. in *Domestication and Exploitation of Plants and Animals* (eds Ucko, P.J. & Dimbleby, G.W.) 355-359 (Aldine, 1969).

SI 3 Evaluation of Possible Link Between Species Ubiquity and Sample Size

The data base for archaeobotanical samples of seeds and fruits from the Bronze Age, Philistine Iron Age Strata, and non-Philistine Iron Age strata examined in this study comprises identifications of 447 plant taxa determined to the species level and below.

To resolve the problem of possible link between sample size and species richness, we ran a Rarefaction test on the plant data base using the PAST statistical package (Hammer et al. 2001). As noted by Magurran (1991), Rarefaction addresses the issue of unequal sample sizes by calculating the numbers of species expected in each sample as if all samples were the same size. This test assumes that the sampled objects are randomly distributed in an environment, they are taxonomically similar and that sampling was carried out in the same manner.

Figure SI 3. Graph showing rarefaction curves for plant remain counts (X axis) by FL against numbers of plant taxa (Y axis).

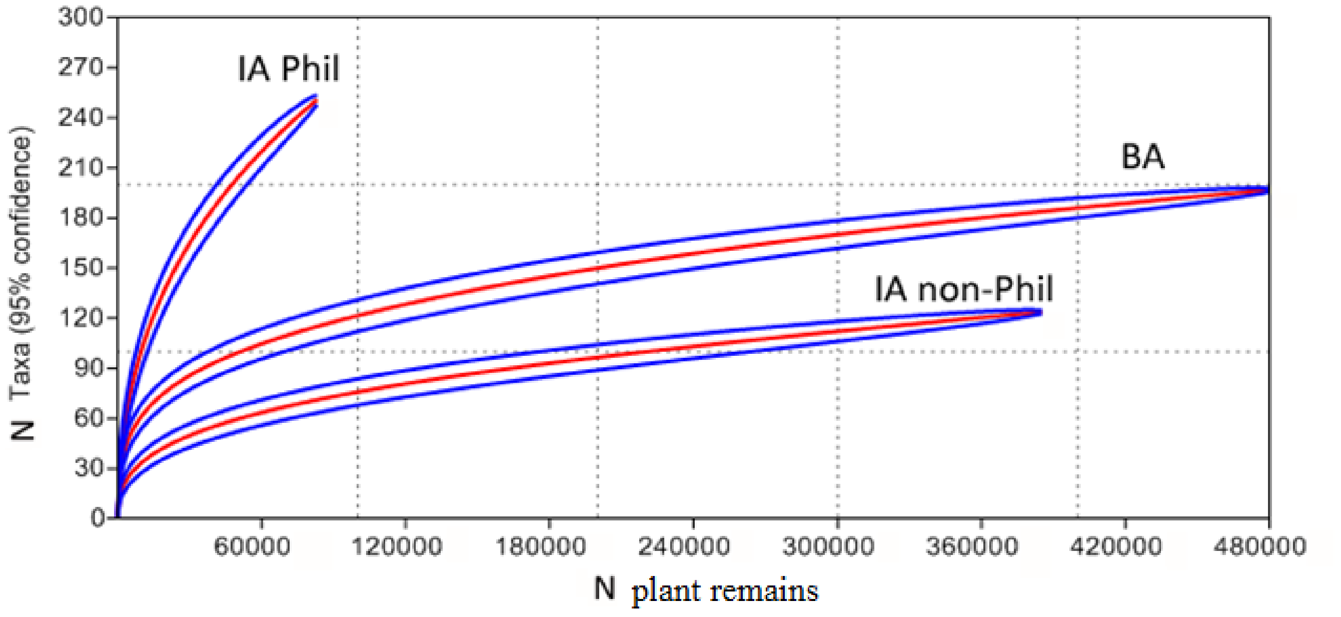

The red line (Fig. SI 3) represents the calculated rarefaction curve for each period, which is a plot of the number of species as a function of the number of specimens (seeds and fruit), while the blue lines represent the 95% confidence limits calculated for each.

The rarefaction curves initially follow a steep slope as the most common species are found. Thereafter they flatten out to form a plateau as the most common species have been sampled and only the rarest species remain to be sampled. As illustrated in the graph, the curves for the Bronze Age and Iron Age non-Philistine FL follow such a pattern. They both flatten out to the right indicating that a reasonable number of individual samples have been examined and that more intensive sampling will yield only few additional species. Moreover, the difference in taxon number between these two samples, indicates that unrelated to sample size, fewer taxa are found in the non-Philistine Iron Age sample.

In contrast, the Iron Age Philistine FL on the left follows a different trajectory, with a steep slope on the left that does not flatten out to the right. This indicates that a large portion of the species diversity still remains to be discovered. While taxon diversity is beginning to flatten out at ca. 60,000 specimens for the two other periods, the curve is still rising for the Iron Age Philistine sample so that it lies far above that for both the Bronze Age and non-Philistine Iron Age FLs.
Clearly, the Iron Age Philistine assemblage contains a significantly higher number of species that will increase further, given a larger sample size. One can extrapolate the trajectory of this curve by extending the line, such that when a sample size of 180,000 specimens is attained, taxon number would reach ca. 300. These results demonstrate that the higher plant diversity in the Iron Age Philistine assemblages is not an artifact of sample size but a true reflection of the composition of the floral assemblage.

References

Hammer Ø., Harper D.A.T. & Ryan P.D. Past: Paleontological Statistics Software Package for Education and Data Analysis. *Palaeontological Electronica* 4 (1), article 4, 9 pp. (2001). http://palaeo-electronica.org/2001_1/pastissue1_01.htm

Magurran A.E. *Ecological Diversity and its Measurement*. 2nd edn. (Chapman and Hall, 1991).
